# Complex association between post-COVID-19 condition and anxiety and depression symptoms

**DOI:** 10.1192/j.eurpsy.2023.2473

**Published:** 2023-12-13

**Authors:** Sarah Tebeka, Laure Carcaillon-Bentata, Valentina Decio, Caroline Alleaume, Nathalie Beltzer, Anne Gallay, Cédric Lemogne, Baptiste Pignon, Tatjana T. Makovski, Joël Coste

**Affiliations:** 1 Department of Non-Communicable Diseases and Injuries, Santé Publique France, The National Public Health Agency, Saint-Maurice, France; 2 Institute of Psychiatry and Neurosciences, Team 1, Université Paris Cité, INSERM UMR1266, Paris, France; 3 Department of Psychiatry, AP-HP, Louis Mourier Hospital, Colombes, France; 4 Center for Research in Epidemiology and Statistics (CRESS), Université Paris Cité and Université Sorbonne Paris Nord, Inserm, INRAE, Paris, France; 5 Service de Psychiatrie de l’Adulte, AP-HP, Hôpital Hôtel-Dieu, Paris, France; 6 DMU IMPACT, INSERM U955, IMRB, Translational Neuropsychiatry, Fondation FondaMental, Université Paris-Est-Créteil (UPEC), AP-HP, Hôpitaux Universitaires “H. Mondor”, Creteil, France

**Keywords:** anxiety, depression, general population, long COVID, persistent symptoms, post-COVID-19 condition

## Abstract

**Background:**

To assess the associations between anxiety and depressive symptoms and post-COVID-19 condition (PCC) by exploring the direction of these associations and their relevance in the definition of PCC.

**Methods:**

Nationwide survey among French adults, recruited between March and April, 2022, using a quota method to capture a representative sample of the general population with regard to sex, age, socioeconomic status, size of the place of residence, and region. We included all participants who met the World Health Organization (WHO) definition of PCC in addition to a random sample of participants infected with SARS-COV-2 for at least 3 months but without PCC. Self-reported anxiety and depressive symptoms, chronic anxiety and depression (for more than 3 years), and anxiety and depression were measured using the GAD-2 and PHQ-2 questionnaires, respectively.

**Results:**

In a sample of 1,095 participants with PCC and 1,021 participants infected with SARS-COV-2 without PCC, 21% had self-reported anxiety and 18% self-reported depression, whereas 33% and 20% had current measured symptoms of anxiety and depression, respectively. The high prevalence of these symptoms cannot only be explained by the characterization of PCC, as only 13.4% of anxiety symptoms and 7.6% of depressive symptoms met the WHO criteria for PCC. Only one participant met the WHO criteria based on self-reported anxiety or depressive symptoms alone, as these were always combined with other symptoms in patients with PCC. Chronic symptoms were associated with PCC (aOR 1.27; 95% CI: 1.00–1.61). In addition, measured anxiety was associated with PCC (aOR = 1.29; 95% CI: 1.02–1.62).

**Conclusions:**

Pre-COVID-19 chronic anxiety and depression may play a role in the development of PCC or share vulnerability factors with it. Our results challenge the inclusion of anxiety and depression in the definition of PCC.

## Introduction

It is now established that SARS-CoV-2 infection can leave patients with persistent symptoms, impaired quality of life, and prolonged suffering, known as “post-COVID-19 condition” (or “long COVID”) [1, 2]. Early prevalence estimates varied considerably depending on the symptoms, follow-up period, and population [4]. In October 2021, the World Health Organization (WHO) proposed a consensus definition to standardize research on this issue [5]. Thus, post-COVID-19 condition (PCC) is defined as the persistence of symptoms 3 months after COVID-19, lasting for at least 2 months, affecting daily functioning, and not being attributable to another diagnosis [5]. The WHO definition is based on a wide range of symptoms, the most common being fatigue, shortness of breath, cough, pain (muscle, joint, chest, headache), altered sense of smell and taste, and attentional disturbances [1–3, 6–8]. Anxiety and depression have also been listed as symptoms that may define PCC [5].

At the same time, the prevalence of anxiety and depressive symptoms and disorders substantially increased between the pre- and mid-pandemic periods in the general population [9, 10] and more intensely in people with COVID-19 [11–16]. It has been suggested that anxiety and depression may play a role in the persistence of certain physical symptoms such as fatigue, sleep disorders, digestive disorders, and pain [17–20]. Indeed, a pre-infection diagnosis of anxiety or depression is a risk factor for persistent symptoms following SARS-CoV-2 infection [21–23], and depression at 1 month of SARS-CoV-2 infection is associated with an increased risk of persistent physical symptoms at 3 months, including pain and dyspnea [24]. Pre-existing anxiety and depressive symptoms or concurrent with SARS-CoV-2 infection could contribute to the development of PCC or share vulnerability factors with it [25].

Conversely, prolonged physical and cognitive symptoms, especially in the context of high uncertainty, can induce anxiety and depressive symptoms [26, 27]. For COVID-19, a high number of acute and subacute complaints was the strongest correlate of mental health deterioration at distance from the acute infection [28]. Therefore, it is unclear whether anxiety and depressive symptoms should be considered PCC symptoms per se rather than the expected consequences of this challenging condition [29].

The objectives of this study are therefore to (i) assess the association between PCC and anxiety and depression, (ii) explore the direction of the association, and (iii) determine the relevance of retaining anxiety and depressive symptoms in the definition of PCC.

## Methods

### Sample

Overall, 27,537 participants from a panel of volunteers living in metropolitan France answered an online questionnaire between March 22 and April 8, 2022, after giving their consent to be included in this research panel, which was developed and maintained by BVA (Paris, France).

Participants were French adults aged 18 years or older selected using a quota sampling method. Thus, the sample had the same sociodemographic characteristics (age, sex, socio-professional category, and region of residence) as the general population based on the National Institute of Statistics and Economic Studies 2016 census. We applied the quotas from this population to calculate individual weights for our statistical analyses.

Our study population consisted of participants with confirmed or probable SARS-CoV-2 infection at least 3 months before the survey, with or without PCC, who completed the mental health assessment (Supplementary Figure S1).

### Variables

#### COVID-19-related information and post-COVID-19 condition

Participants were asked about their infection with SARS-CoV-2, the dates of any other infections, and whether the diagnoses were confirmed with a test. The most recent infection was selected for further analysis (confirmed by a test or not).

Following the WHO definition of PCC, participants had to have a confirmed (positive test) or probable (medical or self-diagnosis without a test) SARS-CoV-2 infection for more than 3 months, while at least one of the symptoms listed by Soriano et al. had to (i) be present and lasting for more than 60 days, (ii) not be present for more than 3 years, (iii) not be explained by an alternative diagnosis, and (iv) have an impact on daily functioning [5].

Confirmed or probable SARS-CoV-2 infection was reported by 10,166 respondents (37.0% of the total population). Of these infected individuals, 3,668 (39.3%) reported having been infected at least 3 months prior to the survey, representing 13.3% of the total surveyed sample.

#### Self-reported anxiety and depressive symptoms

To identify PCC, participants were evaluated for a list of symptoms provided by Soriano et al., which includes anxiety and depressive symptoms. Those who answered “anxiety” or “depression” to the question “Currently or in the last few days, have you suffered from the following symptoms?” were considered to have self-reported anxiety or depressive symptoms, respectively.

To distinguish newly occurred anxiety and depressive symptoms from those of long-lasting disorders, we used the questions from the WHO definition of PCC (“Did this symptom appear more than 3 years ago?”). We considered anxiety or depressive symptoms to be “chronic” if a participant reported the presence of these symptoms for more than 3 years (i.e., pre-COVID-19 condition).

#### Standardized measures of anxiety and depression

Anxiety was measured using the Generalized Anxiety Disorder (GAD)-2 scale, an ultra-short version of the GAD-7 [30, 31]. The GAD-2 consists of two questions: “Over the last two weeks, how often have you been bothered by feeling nervous, anxious, or on edge?” and “Over the last two weeks, how often have you not been able to stop or control worrying?.” Each item was rated on a four-point Likert scale ranging from 0 (“not at all”) to 3 (“nearly every day”). A cut-off of ≥3 points out of 6 has a sensitivity of 65% and a specificity of 88% for anxiety disorders [32].

Depression was measured using the Patient Health Questionnaire (PHQ)-2, an ultra-short version of the PHQ-9 [31, 33]. The PHQ-2 consists of two questions: “Over the last two weeks, how often have you been bothered by little interest or pleasure in doing things?” and “Over the last two weeks, how often have you been feeling down, depressed, or hopeless?”. Each question was rated on a 4-point Likert scale ranging from 0 (“not at all”) to 3 (“nearly every day”). A cut-off of ≥3 points out of 6 has a sensitivity of 83% and a specificity of 90% for major depression [34].

The GAD-2 and PHQ-2 were administered to all subjects with PCC (*N* = 1,095) and to a random sample of participants infected with SARS-COV-2 for at least 3 months but without PCC (*N* = 1,021), henceforth referred to as the “control group.”

#### Other variables of interest

Socio-demographic data included sex, age (18–24, 25–34, 35–44, 45–54, 55–64, ≥65 years), education level (less than secondary, secondary, tertiary short (≤3 years), tertiary long (>3 years)), household size, employment status (paid employment, unemployed, retired, and other inactive), occupation (company manager, entrepreneur; senior manager, professional; middle manager, teacher; office employee; manual worker; no occupation, retired, or studying (inactive)), employer (public, private, self-employed), as well as size and region of residence.

The Minimum European Health Module included in the survey proposes three measures to assess general health [35]. In particular, the second measure is a reliable indicator of chronic conditions (“Do you have any chronic or long-standing illnesses or health problems?”) [36, 37]. The two other measures assess “self-perceived health” and “activity limitations.”

#### Statistical analyses

Data analysis was conducted in several steps.

First, we detailed the prevalence of self-reported anxiety and depression, the chronic nature of these symptoms (i.e., present for more than 3 years), and whether they met the WHO criteria of PCC. We used logistic regression to assess factors associated with self-reported chronic anxiety and depression.

Second, we used the GAD-2 and PHQ-2 scores both as continuous measures of the level of anxiety or depression (GAD-2 and PHQ-2 scores, respectively) and with a cut-off of ≥3/6 (measured anxiety and depression) to identify the presence of an anxiety disorder or major depression, respectively. We calculated the sensitivity and specificity of self-reported symptoms of anxiety and depression compared with these binary measures (measured anxiety and depression). Moreover, we compared the PHQ-2 and GAD-2 scores between the PCC and control groups using linear regression models.

Third, we used logistic regression models to explore the associations between measured anxiety or depression (as outcomes or dependent variables) and PCC (vs. control group) and other variables. Three nested models were successively constructed for each outcome (measured anxiety and depression):

- Model 1: PCC adjusted for socio-demographic variables (sex, age, and education);
- Model 2 was model 1 additionally adjusted for chronic health condition;
- Model 3 was model 2 further adjusted for self-reported chronic anxiety and depressive symptoms.

Interactions were tested for each model using an alpha level of 5% (conventional level).

Finally, in participants with PCC, we performed specific analyses. We compared the anxiety and depression scores (continuous measures) by the length of time since COVID-19 infection. Second, we assessed the correlation between measured anxiety and depression and the number of symptoms on the one hand and the PCC symptoms one by one on the other. Phi coefficients, which are a special case of the Pearson correlation coefficients for binary variables), and *p*-value were reported.

Appropriate sample weights were used in all analyses (descriptive and analytical) to provide valid estimates for the French general population. All statistical analyses were performed using SAS Enterprise version 9.2 (SAS Institute, Cary, NC, USA).

#### Funding source, regulatory approval, and ethics

This research was conducted by the French National Agency for Public Health (Santé Publique France). It did not receive any specific grant from funding agencies in the public, commercial, or not-for-profit sectors.

All participants were given clear information, and consent was systematically obtained by checkboxes, which were necessary to complete the questionnaire.

This survey complies with the French Data Protection Act of January 6, 1978, amended in 2004 and 2018, and the General Regulation on the Protection of Personal Data (RGPD) of April 27, 2016. The data, particularly health data, are collected after obtaining the express consent of the person concerned. The survey was approved by a local ethics committee on April 6, 2022 (CER-Paris-Saclay-2022-041).

## Results

### Participants

**Figure 1.**
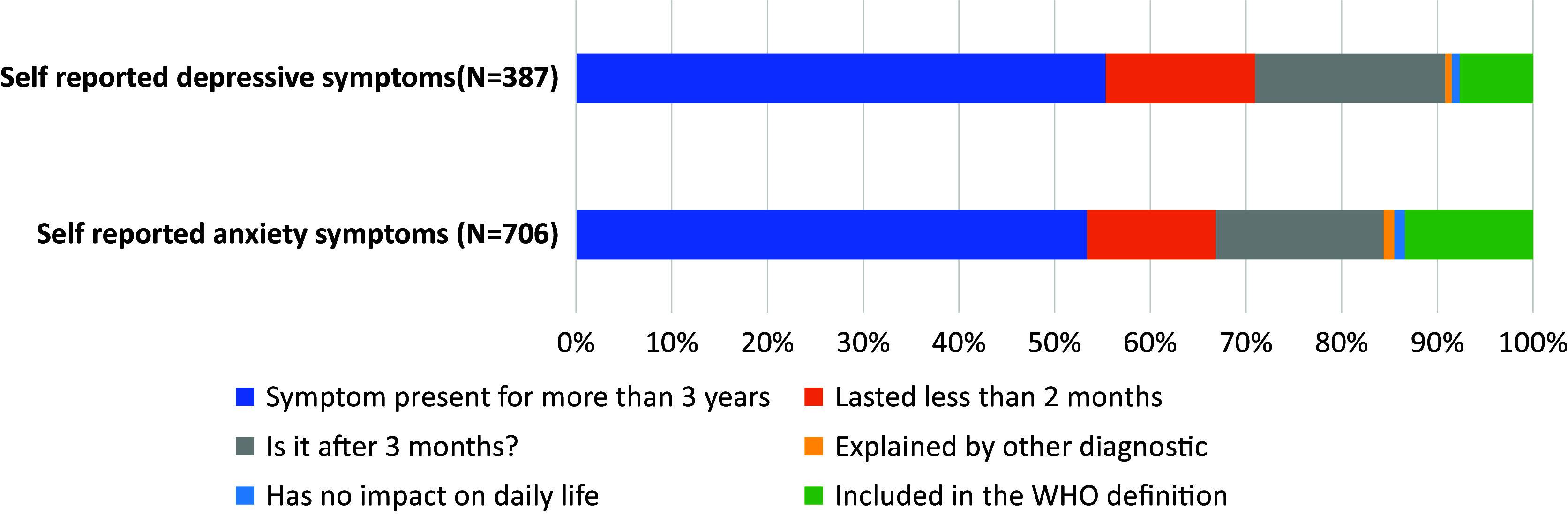
Frequency of self-reported anxiety and depressive symptoms in SARS-COV-2 infected subjects, and criteria for excluding these symptoms in the WHO definition of post-COVID-19 condition.

A total of 1,095 participants with PCC and 1,021 with SARS-COV-2 infection for at least 3 months without PCC (i.e., control group) had a complete health evaluation and were included in this study (*N* = 2,116, Figure 1). The PCC and control groups shared common characteristics: they were rather young (respectively 37.5% and 34.8% under 34), lived alone (81.7% and 82.3%), and were employed in the private (41.8% and 37.4%) or public (24.9% and 22.5%) sector (Table 1).

**Table 1.** Characteristics of the survey sample according to two groups: post-COVID-19 condition and confirmed or probable SARS-COV-2 infection at least 3 months prior to the survey without post-COVID-19 condition

	Post-COVID-19 condition		SARS-COV-2 infection at least 3 months prior to the survey (without post-COVID condition)	
	1,095		1,021	
	*N*	*%*	*N*	*%*
Sex				
Men	431	39	492	48.2
Women	664	60.6	529	51.8
Age				
18–24 years	107	9.8	93	9.2
25–34 years	304	27.8	262	25.7
35–44 years	213	19.5	175	17.1
45–54 years	194	17.7	183	17.9
55–64 years	153	14.0	161	15.8
≥65 years	124	11.3	147	14.4
Education				
Less than secondary	217	19.8	230	22.6
Secondary	242	22.1	222	21.7
Tertiary short ≤ 3 years	392	35.8	364	35.7
Tertiary long >3 years	244	22.3	205	20.1
Household size				
1 person	895	81.7	840	82.3
2+ persons	200	18.3	181	17.7
Employment status				
Paid employment	788	72.0	684	67.0
Unemployed	39	3.6	48	4.7
Retired	243	22.2	271	26.6
Other inactive	25	2.3	18	1.8
Occupation				
Company manager, entrepreneur	33	3.0	37	3.6
Senior manager, professional	232	21.2	206	20.1
Middle manager, teacher	162	14.8	140	13.7
Office employee	370	33.8	318	31.1
Manual worker	30	2.8	31	3.0
No occupation, retired, or studying (inactive)	268	24.4	289	28.3
Employer				
Public sector	273	24.9	230	22.5
Private sector	458	41.8	382	37.4
Self-employed	57	5.2	72	7.1
Other	307	28.0	337	33.0
Size of place of residence				
Less than 20,000 inhabitants	364	33.2	361	35.3
20,000–1,999,999 habitants	510	46.6	439	43.0
City of Paris	221	20.2	221	21.7
Region				
Ile-de-France (Greater Paris)	247	22.6	253	24.8
North-East	270	24.7	221	21.7
North-West	169	15.4	162	15.8
South-East	273	24.9	240	23.5
* South-West*	136	12.4	145	14.2
Minimum European Health Module (MEHM)				
Self-perceived health				
Good to very good	471	43.0	589	57.7
Fair	457	41.7	331	32.4
Bad to very bad	167	15.3	101	9.9
Chronic condition				
Yes	455	41.6	408	39.9
No	640	58.4	613	60.1
Activity limitations				
Severely limited	115	10.5	87	8.5
Limited but not severely	355	32.5	234	22.9
Not limited at all	625	57.0	700	68.6

Regarding health status, PCC participants more often reported a bad to very bad self-perceived health compared with the control group (15.3% vs. 9.9%). They also reported slightly more chronic conditions (41.6% vs. 39.9%) and activity limitations (43.0% vs. 31.4%) (Table 1).

### Self-reported anxiety and depressive symptoms

#### Prevalence

Self-reported anxiety and depressive symptoms concerned 33.4% and 18.0% of our study sample, respectively. Overall, 13.4% and 7.6% of participants reporting anxiety and depressive symptoms, respectively, also met the WHO criteria for PCC (Figure 1). We observed that over half of the subjects with self-reported anxiety and depressive symptoms (53.4% and 55.4%, respectively) had chronic symptoms.

Self-reported anxiety and depressive symptoms were rarely isolated symptoms in patients with PCC: when present, they were systematically associated with other symptoms meeting the definition of PCC. Among 1,095 participants with PCC, only one had self-reported anxiety as the only symptom, and none had self-reported depression as the only symptom.

#### Self-reported chronic anxiety and depressive symptoms

Chronic anxiety and depressive symptoms (i.e., present for more than 3 years) were found in 18.4% and 10.7% of PCC participants, respectively, versus 14.8% and 9.2% of the control group participants. Chronic anxiety symptoms were significantly associated with PCC (aOR = 1.27; 95% CI: 1.00–1.61, *p*-value = 0.04) after adjustment for sex and age (Supplementary Table S1). Chronic depressive symptoms were not associated with PCC.

### Standardized measures of anxiety and depression

#### Measured anxiety and depression

Measured anxiety (GAD-2 score ≥ 3) and depression (PHQ-2 score ≥ 3) concerned 20.5 and 20.1% of all participants, respectively. Using these binary measures as a reference, the sensitivity/specificity of self-reported anxiety and depressive symptoms were 64.5%/74.5 and 52.8%/90.2%, respectively (Supplementary Tables S2 and S3).

Measured anxiety concerned 23.3% (95% CI: 20.8–25.8) of PCC participants versus 17.6% (95% CI: 15.2–19.2) in the control group. Measured depression concerned 22.2% (95% CI: 19.7–24.6) of PCC participants versus 18.0% (95% CI: 15.6–20.4) in the control group.

**Figure 2.**
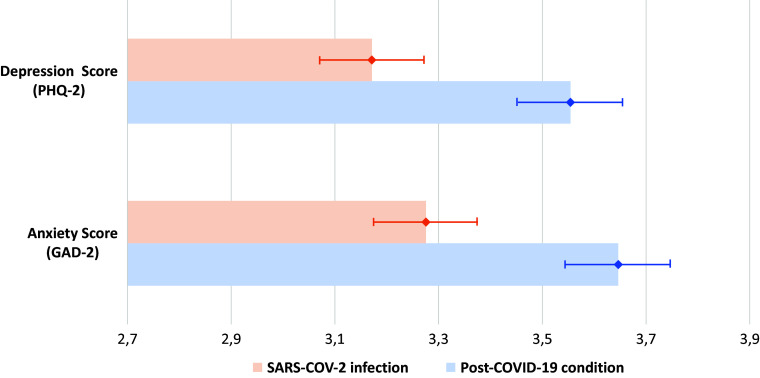
Measured depression and anxiety scores among SARS-COV-2 infected participants with and without post-COVID-19 condition.

Anxiety (GAD-2 score) and depression levels (PHQ-2 score) at the time of the evaluation were significantly higher in the PCC group than in the control group (mean GAD-2 score: 3.65 vs. 3.28, *p* < 0.0001; mean PHQ2 score: 3.55 vs. 3.17, *p* < 0.0001) (Figure 2).

#### Factors associated with measured anxiety and depression

In bivariate analysis, measured anxiety (GAD-2 score ≥ 3) was associated with PCC, female sex, and having another chronic condition, whereas age ≥ 65 years was a protective factor (Table 2). In multivariate analysis, measured anxiety was associated with PCC after adjustment for socio-demographic data (model 1: aOR = 1.34 95% CI: 1.08–1.67). This association remained significant, without attenuation, after successively adjusting for the chronic condition (model 2: aOR = 1.32, 95% CI: 1.06–1.65) and for self-reported chronic anxiety (model 3: aOR = 1.29, 95% CI: 1.02–1.62), which was nonetheless the strongest predictor of measured anxiety (Model 3: aOR = 4.43, 95% CI: 3.43–5.71).

**Table 2.** Factors independently associated with measured anxiety (GAD-2) and depression (PHQ-2)

	Measured anxiety									Measured depression								
	Model 1			Model 2			Model 3			Model 1			Model 2			Model 3		
	aOR	95% CI		aOR	95% CI		aOR	95% CI		aOR	95% CI		aOR	95% CI		aOR	95% CI	
COVID-19 status																		
Post-COVID-19 condition	**1.34**	1.08	1.67	**1.32**	1.06	1.65	**1.29**	1.02	1.62	1.22	0.98	1.52	1.20	0.96	1.50	1.19	0.95	1.51
SARS-COV-2 infection	Ref			Ref			Ref			Ref			Ref			Ref		
Sex																		
Men	Ref			Ref			Ref			Ref			Ref			Ref		
Women	**1.72**	1.36	2.18	**1.63**	1.28	2.07	**1.63**	1.28	2.09	**1.48**	1.17	1.87	**1.38**	1.09	1.75	**1.37**	1.07	1.76
Age																		
18–24 years	1.11	0.76	1.62	1.10	0.75	1.61	1.06	0.72	1.57	1.40	0.97	2.02	1.40	0.96	2.02	1.33	0.90	1.97
25–34 years	Ref			Ref			Ref			Ref			Ref			Ref		
35–44 years	0.79	0.57	1.09	0.74	0.53	1.03	0.80	0.57	1.12	0.98	0.72	1.35	0.92	0.67	1.27	0.92	0.66	1.29
45–54 years	1.00	0.72	1.38	0.89	0.64	1.24	0.89	0.63	1.26	**0.68**	0.48	0.95	**0.58**	0.41	0.82	**0.57**	0.40	0.82
55–64 years	0.85	0.59	1.22	0.70	0.48	1.01	0.77	0.52	1.12	0.71	0.49	1.02	**0.55**	0.38	0.80	**0.57**	0.39	0.85
≥65 years	**0.42**	0.27	0.68	**0.31**	0.19	0.51	**0.35**	0.21	0.59	**0.48**	0.31	0.75	**0.33**	0.21	0.53	**0.38**	0.24	0.60
Education																		
Less than secondary																		
Secondary	0.96	0.67	1.36	0.99	0.69	1.41	0.96	0.66	1.40									
Tertiary short ≤ 3 years	1.17	0.85	1.60	1.23	0.89	1.68	1.27	0.91	1.78									
Tertiary long >3 years	1.38	0.97	1.95	**1.44**	1.01	2.04	**1.52**	1.05	2.19									
Chronic condition																		
No				Ref														
Yes				**1.85**	1.46	2.33	**1.62**	1.27	2.06				**2.10**	1.66	2.65	**1.82**	1.42	2.33
Mental health history																		
Self-reported chronic anxiety							**4.43**	3.43	5.71									
Self-reported chronic depression																**7.03**	5.14	9.61

Abbreviations: aOR, adjusted odd-ratio; CI, confidence interval.

Model 1: Adjusted for sex, age, and education.

Model 2: Adjusted for sex, age, education, and chronic condition.

Model 3: Adjusted for sex, age, education, chronic condition, and self-reported chronic anxiety (for anxiety) or self-reported chronic depression (for depression).

Significant differences are in bold.

There was no significant association between measured depression (PHQ-2 score ≥ 3) and PCC (Table 2). Factors positively associated with measured depression were self-reported chronic depression (model 3: aOR = 7.03, 95% CI: 5.14–9.61), the presence of another condition, and female sex. Age > 45 years was a protective factor. Interactions were not significant in any model.

#### Length of time since SARS-COV-2 infection in participants with post-COVID-19 condition and anxiety or depression

Among PCC participants, the length of time since SARS-COV-2 infection was not associated with levels of anxiety or depression (GAD-2 and PHQ-2 scores, respectively) (Supplementary Figure S2). The results were similar for participants without PCC (data not shown).

#### Correlation between anxiety or depression and number of post-COVID-19 condition symptoms

**Figure 3.**
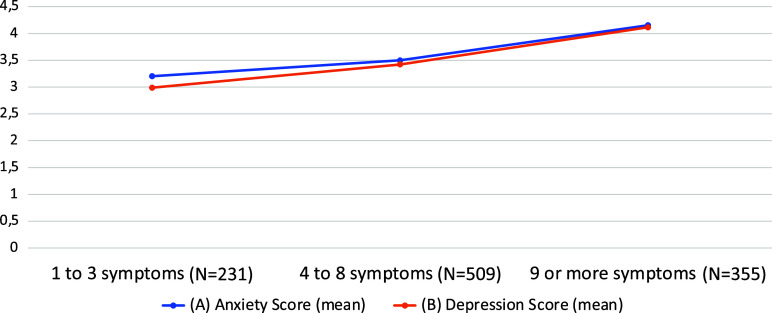
Relationship between the number of symptoms and (A) measured anxiety and (B) measured depression in post-COVID-19 condition participants.

Among PCC participants, levels of anxiety and depression (GAD-2 and PHQ-2 scores, respectively) were both strongly correlated with the number of PCC symptoms (correlation coefficients 0.24 and 0.27, respectively, *p* < 0.001) (Figure 3). More specifically, levels of anxiety (GAD-2 score) were strongly correlated with depression (0.40, *p* < 0.001), cognitive disorders (0.24, *p* < 0.001), sleep disorders (0.22, *p* < 0.001), and blurred vision (0.19, *p* < 0.001). Levels of depression (PHQ-2 score) were strongly correlated with anxiety symptoms (0.40, *p* < 0.001), cognitive disorders (0.26, *p* < 0.001), paresthesia (0.22, *p* < 0.001), and sleep disorders (0.21, *p* < 0.001) (Supplementary Tables S4 and S5).

## Discussion

In this nationwide survey, we examined the prevalence and correlates of anxiety and depressive symptoms in participants infected with SARS-CoV-2 with and without PCC.

Anxiety and depression were very common symptoms after SARS-COV-2 infection, especially in subjects with PCC (prevalence of measured anxiety/depression of 23.3%/22.2% for PCC participants vs. 17.6%/18.0 in the control group). Our results are consistent with a recent meta-analysis of persistent symptoms 1 year after COVID-19 infection, which found a prevalence of anxiety symptoms of 22% (95% CI: 15–29) and depression of 23% (95% CI: 12–34) [38].

Although anxiety and depression were frequent symptoms, they were almost never the only PCC symptoms, as they were systematically associated with other symptoms in all but one participant with PCC. These findings challenge the inclusion of anxiety and depression in the list of eligible symptoms for diagnosing PCC.

Chronic self-reported anxiety (i.e., present before the COVID-19 pandemic) was significantly more frequent in the PCC group compared with the control group, which is consistent with the identification of pre-infection psychiatric comorbidities as risk factors for PCC [22, 23, 39]. These data support the broader literature linking anxiety and depression with the occurrence or persistence of physical symptoms [17–20], thus suggesting possible causal pathways and shared vulnerability factors.

Participants with PCC had higher levels of anxiety and depressive symptoms than participants infected with SARS-COV-2 infection without PCC. The association between measured anxiety and PCC remained significant after adjusting for socio-demographic data, chronic condition, and chronic anxiety. Interestingly, risk factors for measured anxiety and depression were similar to those for PCC. Indeed, female sex and chronic conditions are both well-established risk factors for PCC [40–45]. These results reinforce the hypothesis of a shared vulnerability between these psychiatric manifestations and PCC. Age-related data are more controversial: while some studies show that young people are at greater risk of developing PCC [39, 46], others found an association between increasing age and PCC [40, 42, 44, 47, 48]. These discrepancies might be due to different between-sample rates of severe COVID-19, which are more frequent in older people.

For participants both with and without PCC, time since SARS-COV-2 infection did not affect the level of measured anxiety or depression. Several studies have examined the course of PCC symptoms. For the majority of patients, the number of symptoms decreased over time [49–51], even though a worsening of neurocognitive symptoms has been reported [52]. The lack of association between anxiety and depression symptoms and the time of infection has already been observed after severe COVID-19 [53], which is consistent with the view that anxiety and depression are contributing factors or comorbidities rather than mere consequences of the disabling symptoms of PCC.

Levels of both anxiety and depression were particularly correlated with PCC symptoms, which are also diagnostic criteria for anxiety or depressive disorders such as cognitive impairment and sleep disorders. This is consistent with a study that found three clusters of PCC symptoms, one of which was dominated by depression, anxiety, insomnia, and “brain fog” [39]. However, this was not true for fatigue, a core symptom of PCC, which was weakly correlated with anxiety and depression in our study. Finally, we found that levels of anxiety and depression were both associated with the number of total symptoms (i.e., the burden of PCC), which is consistent with a possible causal relationship.

This study has several strengths. First, it is based on a large population-based sample, which assesses the extent of a problem at the population level, as opposed to hospital settings in which most studies on PCC have been conducted to date [11, 47, 51]. A population-based approach provides crucial information for public health decisions. Second, we used a controlled design, which allowed us to compare participants with PCC to those infected with SARS-COV-2 who did not develop PCC. Third, we favored the WHO definition to have external validity and ensure the comparability of our work. However, while this consensual definition has many advantages, it is open to criticism, in particular concerning the inclusion of non-specific symptoms, the default attribution to SARS-CoV-2 infection of any otherwise unexplained symptoms, and the lack of recognition of one’s perception of long COVID (i.e., self-reported long COVID) in patients which endure prolonged symptoms but which are not necessarily captured by the PCC definition [54]. In addition, the WHO definition has some evasive criteria that are difficult to translate into research practice and may lead to studies’ specific adaptation. This may hamper the comparability between studies using this same definition. Finally, we assessed anxiety and depressive symptoms using both self-reported and standardized measures to obtain a more comprehensive view. Each of these measures has its biases that may downward the rates: on the one hand, the self-reported measures of chronic anxiety and chronic depression are subject to under-assessment; on the other hand, the short versions of the GAD and PHQ are less sensitive than the long versions.

Several limitations should nevertheless be acknowledged. First, the observational and cross-sectional design of this study does not allow for causal or directional conclusions. Second, the quota sampling methodology based on a panel of volunteers may limit the generalization of our results. Finally, we did not consider the severity of the initial COVID-19 episode. However, it has been shown that severe SARS-COV-2 infection, marked by an increased number of symptoms in the acute phase or by hospitalization, is associated with anxiety and depressive disorders [55–57].

## Conclusion

This work highlights the complex association between mental health and PCC, especially the potential role played by chronic psychiatric symptoms, and in particular chronic anxiety, in the risk of developing PCC. Although our data do not allow to conclude any causal relation, further studies may examine potential causal pathways and shared vulnerability factors, as such knowledge may inform preventive strategies. Finally, our results challenge the inclusion of anxiety and depression symptoms in the definition of PCC.

## Supporting information

Tebeka et al. supplementary material 1 Tebeka et al. supplementary material

Tebeka et al. supplementary material 2 Tebeka et al. supplementary material

Tebeka et al. supplementary material 3 Tebeka et al. supplementary material

Tebeka et al. supplementary material 4 Tebeka et al. supplementary material

Tebeka et al. supplementary material 5 Tebeka et al. supplementary material

## Data Availability

The data that support the findings of this study are available from Santé Publique France. Restrictions apply to the availability of these data, which were used under license for this study. Data are available from the authors with the permission of Santé Publique France.

## Supplementary material

The supplementary material for this article can be found at https://doi.org/10.1192/j.eurpsy.2023.2473.
