# Supplementary material for: Complex association between post-COVID-19 condition and anxiety and depression symptoms
Source: Eur Psychiatry. 2023 Dec 13;67(1):e1. doi: 10.1192/j.eurpsy.2023.2473 (PMC10964277; doi:10.1192/j.eurpsy.2023.2473)
Supplement: Tebeka et al. supplementary material 1 — Tebeka et al. supplementary material [file S0924933823024732sup001.pptx]

## Slide 1
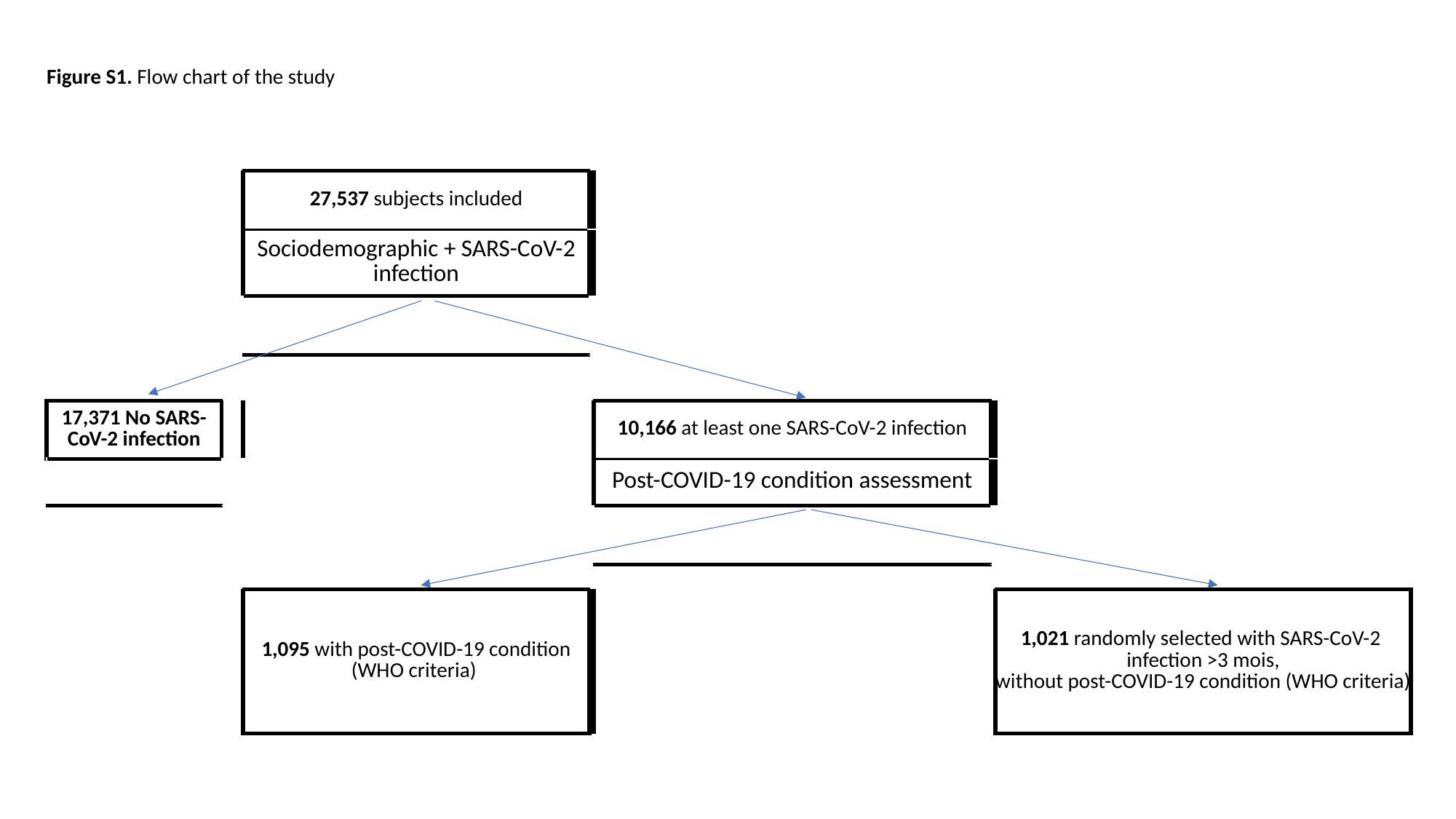

| Figure S1. Flow chart of the study | | | | | | |
| --- | --- | --- | --- | --- | --- | --- |
| | | | | | | |
| | | 27,537 subjects included | | | | |
| | | Sociodemographic + SARS-CoV-2 infection | | | | |
| | | | | | | |
| | | | | | | |
| | | | | | | |
| 17,371 No SARS-CoV-2 infection | | | | 10,166 at least one SARS-CoV-2 infection | | |
| | | | | Post-COVID-19 condition assessment | | |
| | | | | | | |
| | | | | | | |
| | | 1,095 with post-COVID-19 condition (WHO criteria) | | | | 1,021 randomly selected with SARS-CoV-2 infection >3 mois, without post-COVID-19 condition (WHO criteria) |
