# Supplementary material for: Complex association between post-COVID-19 condition and anxiety and depression symptoms
Source: Eur Psychiatry. 2023 Dec 13;67(1):e1. doi: 10.1192/j.eurpsy.2023.2473 (PMC10964277; doi:10.1192/j.eurpsy.2023.2473)
Supplement: Tebeka et al. supplementary material 2 — Tebeka et al. supplementary material [file S0924933823024732sup002.pptx]

## Slide 1
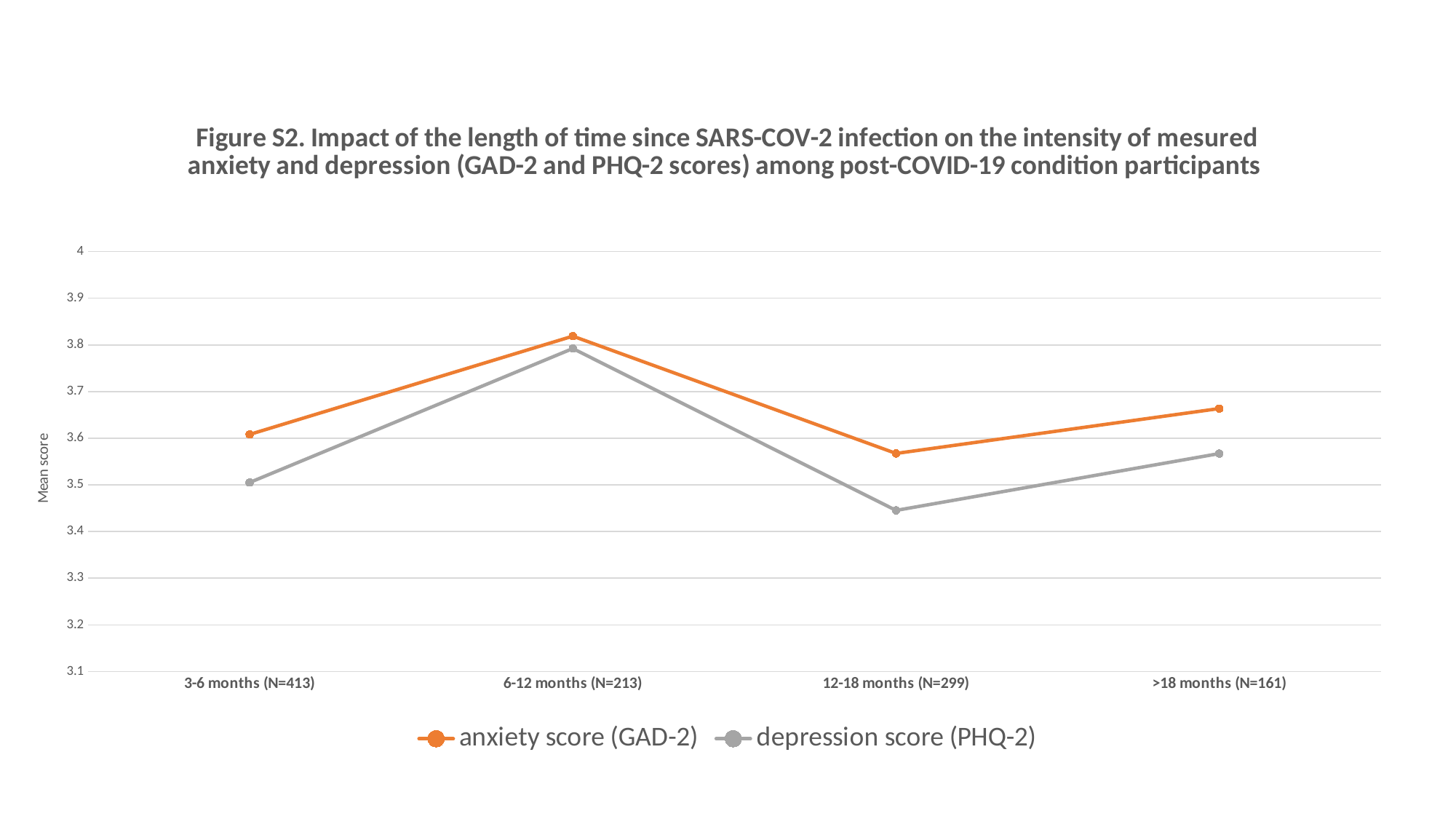

### Chart: Figure S2. Impact of the length of time since SARS-COV-2 infection on the intensity of mesured anxiety and depression (GAD-2 and PHQ-2 scores) among post-COVID-19 condition participants
| Category | anxiety score (GAD-2) | depression score (PHQ-2) |
|---|---|---|
| 3-6 months (N=413) | 3.608047 | 3.50505 |
| 6-12 months (N=213) | 3.81872 | 3.792141 |
| 12-18 months (N=299) | 3.5673 | 3.445273 |
| >18 months (N=161) | 3.663608 | 3.567078 |
