# Supplementary material for: Complex association between post-COVID-19 condition and anxiety and depression symptoms
Source: Eur Psychiatry. 2023 Dec 13;67(1):e1. doi: 10.1192/j.eurpsy.2023.2473 (PMC10964277; doi:10.1192/j.eurpsy.2023.2473)
Supplement: Tebeka et al. supplementary material 3 — Tebeka et al. supplementary material [file S0924933823024732sup003.docx]

| **Table S1.** Factors independently associated with self-reported chronic anxiety and chronic depressive symptoms | | | | | |  |
| --- | --- | --- | --- | --- | --- | --- |
|  | **Chronic anxiety symptoms** | | | **Chronic depressive symptoms** | | |
|  | aOR | 95% CI | | aOR | 95% CI | |
| *COVID-19 status* |  |  |  |  |  |  |
| Post-COVID-19 condition | **1,27** | **1.00*** | **1,61** | 1,14 | 0,85 | 1,52 |
| SARS-COV-2 infection | ref |  |  | ref |  |  |
| *Sex* |  |  |  |  |  |  |
| Men | ref |  |  | ref |  |  |
| Women | 1,26 | 0,98 | 1,61 | 1,31 | 0,96 | 1,79 |
| *Age* |  |  |  |  |  |  |
| 18-24 years | 1,19 | 0,79 | 1,78 | 1,38 | 0,85 | 2,25 |
| 25-34 years | ref |  |  | ref |  |  |
| 35-44 years | 0,76 | 0,53 | 1,09 | 1,03 | 0,67 | 1,57 |
| 45-54 years | 1,09 | 0,77 | 1,53 | 1,00 | 0,65 | 1,55 |
| 55-64 years | 0,85 | 0,58 | 1,24 | 0,87 | 0,54 | 1,42 |
| ≥ 65 years | 0,65 | 0,42 | 1,02 | 0,56 | 0,31 | 1,03 |
|  |  |  |  |  |  |  |
| abreviations: aOR = adjusted odd-ratio | | |  |  |  |  |
| * 1.003, p-value =0.04 |  |  |  |  |  |  |
| Significant differences are in bold. | |  |  |  |  |  |
