# Supplementary material for: Complex association between post-COVID-19 condition and anxiety and depression symptoms
Source: Eur Psychiatry. 2023 Dec 13;67(1):e1. doi: 10.1192/j.eurpsy.2023.2473 (PMC10964277; doi:10.1192/j.eurpsy.2023.2473)
Supplement: Tebeka et al. supplementary material 4 — Tebeka et al. supplementary material [file S0924933823024732sup004.docx]

| **Table S2.** Concordance of anxiety measures  (anxiety assessed by the GAD-2 and self-reported anxiety) | | | |
| --- | --- | --- | --- |
|  |  | **Measured anxiety (GAD-2)** | |
|  |  | Yes | No |
| **Self-reported anxiety** | Yes | 278 (13%) | 429 (20%) |
|  | No | 157 (7%) | 1253 (59%) |
| **Chronic self-reported anxiety** | Yes | 164 (8%) | 189 (9%) |
|  | No | 271 (13%) | 1493 (71%) |
|  |  |  |  |
| **Table S3.** Concordance of depressive symptoms (depression assessed by the PHQ-2 and self-reported depression) | | | |
|  |  | **Measured depression (PHQ-2)** | |
|  |  | Yes | No |
| **Self-reported depressive symptoms** | Yes | 225 (11%) | 165 (8%) |
|  | No | 201 (10%) | 1524 (72%) |
| **Chronic self-reported depressive symptoms** | Yes | 126 (6%) | 86 (4%) |
|  | No | 300 (14%) | 1604 (76%) |
|  |  |  |  |
