# Supplementary material for: Complex association between post-COVID-19 condition and anxiety and depression symptoms
Source: Eur Psychiatry. 2023 Dec 13;67(1):e1. doi: 10.1192/j.eurpsy.2023.2473 (PMC10964277; doi:10.1192/j.eurpsy.2023.2473)
Supplement: Tebeka et al. supplementary material 5 — Tebeka et al. supplementary material [file S0924933823024732sup005.docx]

| **Table S4.** Correlation (phi coefficients) between mesured anxiety score and other symptoms reported by participants with post-COVID-19 condition (N= 1,095) | |  | **Table S5.** Correlation (phi coefficients) between mesured depression score and other symptoms reported by participants with post-COVID-19 condition (N= 1,095) | |
| --- | --- | --- | --- | --- |
| **Self-declared symptoms** | **Mesured anxiety (GAD-2 score)** |  | **Self-declared symptoms** | **Mesured depression (PHQ-2 score)** |
| Depression | 0.40 |  | Anxiety | 0.40 |
| Cognitive dysfunction/ impaired memory | 0.24 |  | Cognitive dysfunction/impaired memory | 0.26 |
| Sleep disorders | 0.22 |  | Pins and needles sensations | 0.22 |
| Blurred vision | 0.19 |  | Sleep disorders | 0.21 |
| Abdominal pain | 0.17 |  | Blurred vision | 0.20 |
| Dizziness/post-exertional malaise | 0.17 |  | Dizziness/post-exertional malaise | 0.19 |
| Pins and needles sensations | 0.17 |  | Tachycardia | 0.17 |
| Tachycardia | 0.17 |  | Abdominal pain | 0.16 |
| Altered menstruation | 0.17 |  | Neuralgia | 0.16 |
| New onset allergies | 0.14 |  | Chest pain | 0.16 |
| Neuralgia | 0.13 |  | New onset allergies | 0.14 |
| Tinnitus | 0.13 |  | Muscle spasms | 0.14 |
| Chest pain | 0.12 |  | Altered menstruation | 0.12 |
| Headache | 0.11 |  | Joint pain | 0.12 |
| Fatigue | 0.09 |  | Tinnitus | 0.12 |
| Joint pain | 0.07 |  | Fatigue | 0.11 |
| Altered smell | -0.07 |  | Headache | 0.09 |
| Muscle spasms | 0.06 |  | Shortness of breath | 0.07 |
| Shortness of breath | 0.05 |  | Altered smell | -0.04 |
| Altered taste | -0.05 |  | Altered taste | -0.03 |
| Cough | 0.01 |  | Fever | 0.02 |
| Fever | 0.01 |  | Cough | 0.01 |
